# All Wrong

**Published:** 1885-07

**Authors:** Thos. Herbert

**Affiliations:** New Iberia, La.


### ALL WRONG.

Dr. Thomas Herbert, of New Iberia, La., has a very ingenious, and well-written article, in the *New Orleans Medical and Surgical Journal*, for June, on the "Question of Clothing."

He states the proposition, which is generally taught and accepted, that (as a rule) white reflects, and black absorbs heat; that, therefore, white is a non-conductor, and black a conductor; that white, worn next to the body, will prevent the escape of the heat of the body, and thus prove hot in summer, and warm in winter. We will state, parenthetically, that, from these remarks, we may infer, the Doctor never had occasion to get in between a pair of linen sheets in midwinter, or he would have discovered with what rapidity white conducts heat—away!

Starting from the above premises the Doctor argues, that we are going contrary to nature in wearing white in summer, and black in winter, and says that it is all a mistake about white linen being cooler in summer than black woolen next to our persons. He cites, as an illustration, (and says that nature makes no mistakes) that the inhabitants are blackest nearest the equator—the negroes and the bears; and that the polar bear is always white. If black intensifies heat, and white intensifies cold, nature, he argues, has been cruel in that arrangement, and it should be reversed; ergo, we, to acquire the greatest amount of comfort from our clothing, should imitate nature; reverse the order of our dress, and wear white [duck suits?] in winter, and black alpaca and flannel next the skin in summer. He cites that nature's garments are white, pure white—the snow—in winter, and says ours should be. Does the snow not keep the ground warm, and cause the wheat to sprout beneath? The Doctor goes farther, and says, it is all nonsense about a white straw hat being cool in summer; "it would not be half as cool as a bar of iron (a good conductor) would be."

Imagine a man going about the streets of New Orleans to-day with an iron hat on!

Now, there is some sense in the Doctor's article, but he seems to have forgotten that "circumstances alter cases." When the heat of the body is greater than the surrounding atmosphere, (as in winter) a conductor of heat would carry off the heat of the body, and thus prove cold. When the heat is greater in the surrounding atmosphere than it is in the body, (as it is in the summer sun) a conductor would carry the heat *to* the body, and thus prove uncomfortably hot to the wearer. This is the explanation why a bar of iron is very cold in winter, and the reverse in the summer sun.

There is no such property or quality in matter as cold; heat is resident in, and common to, all matter. We estimate the temperature of a body by the increase or diminution of heat, and by the rapidity with which a given body abstracts heat from the part brought in contact with it. Iron is not really cold in winter, but it rapidly abstracts the heat from the hand when touched, and thus gives the impression of being very cold.

In the Carre process for making ice, the principle is, the affinity which vaporized ammonia has for caloric; it abstracts the heat from the water, and leaves the water frozen. Thus Doctor Herbert would find his iron hat *very cold* in winter—only.

But, who knows if the Doctor is not entirely right, after all? and that the world has been going wrong all this while; and now, that he has called attention to it, like a modern Gallilleo, there may be a complete revolution, a breaking up of old ideas, an abandonment of old habits, and an era of dressing on sound scientific principles established, whereby all diseases, incidental to the errors of dress, like "taking cold" for instance, will be abolished? Did the world not wag on for many centuries content, under the fallacious belief, that the eye should be in the big end of the needle? Did not our grand-mothers spend their lives content to sew with such needles, and were blissful in their ignorance, till here comes along a philosopher, who demonstrates that it is all a mistake, and puts the hole through the *point*, and, presto, the sewing machine is born? What a revelation has taken place? and the manufacturing world has been revolutionized.

But *how* odd it will appear, for awhile, to see everybody running about in the snow, dressed in white duck, and draped in sombre robes, like the Nuns, when the thermometer is having a series of acrobatic performances up among the cross-bars of the "nineties in the shade!"

